# Cellular Response to Bone Morphogenetic Proteins-2 and -7 Covalently Bound to Photocrosslinked Heparin–Diazoresin Multilayer

**DOI:** 10.3390/biom13050842

**Published:** 2023-05-15

**Authors:** Magdalena Wytrwal, Małgorzata Sekuła-Stryjewska, Agata Pomorska, Ewa Oclon, Ewa Zuba-Surma, Szczepan Zapotoczny, Krzysztof Szczubiałka

**Affiliations:** 1 Academic Centre for Materials and Nanotechnology, AGH University of Science and Technology, al. A. Mickiewicza 30, 30-059 Krakow, Poland; zapotocz@agh.edu.pl; 2 Malopolska Centre of Biotechnology, Jagiellonian University, Gronostajowa 7A, 30-387 Krakow, Poland; malgorzata_sekula@wp.pl; 3 Jerzy Haber Institute of Catalysis and Surface Chemistry, Polish Academy of Sciences, Niezapominajek 8, 30-239 Krakow, Poland; agata.pomorska@ikifp.edu.pl; 4 Laboratory of Recombinant Proteins Production, Centre for Experimental and Innovative Medicine, University of Agriculture in Krakow, 1C Redzina Street, 30-248 Krakow, Poland; ewa.oclon@urk.edu.pl; 5 Faculty of Biochemistry, Biophysics and Biotechnology, Jagiellonian University, Gronostajowa 7, 30-387 Krakow, Poland; ewa.zuba-surma@uj.edu.pl; 6 Faculty of Chemistry, Jagiellonian University, Gronostajowa 2, 30-387 Krakow, Poland; szczubia@chemia.uj.edu.pl

**Keywords:** bone morphogenetic protein-2, bone morphogenetic protein-7, cell culture surfaces, heparin, diazoresin

## Abstract

Despite the plethora of research that exists on recombinant human bone morphogenetic protein-2 and -7 (rhBMP-2 and rhBMP-7) and has been clinically approved, there is still a need to gain information that would allow for their more rational use in bone implantology. The clinical application of supra-physiological dosages of these superactive molecules causes many serious adverse effects. At the cellular level, they play a role in osteogenesis and cellular adhesion, migration, and proliferation around the implant. Therefore, in this work, we investigated the role of the covalent binding of rhBMP-2 and rhBMP-7 separately and in combination with ultrathin multilayers composed of heparin and diazoresin in stem cells. In the first step, we optimized the protein deposition conditions via quartz crystal microbalance (QCM). Then, atomic force microscopy (AFM) and enzyme-linked immunosorbent assay (ELISA) were used to analyze protein–substrate interactions. The effect of the protein binding on the initial cell adhesion, migration, and short-term expression of osteogenesis markers was tested. In the presence of both proteins, cell flattening and adhesion became more prominent, resulting in limited motility. However, the early osteogenic marker expression significantly increased compared to the single protein systems. The presence of single proteins resulted in the elongation of cells, which promoted their migration activity.

## 1. Introduction

Growth factors are a powerful class of cell signaling molecules [1]. They are secreted by cells and control many cellular responses, e.g., proliferation, migration, and differentiation. The design and production of new materials for bone engineering is a broad research area where the use of growth factors, especially bone morphogenetic proteins (BMPs), is particularly important. A vast amount of research focuses on the usage of BMP-2 and BMP-7, as they are authorized by the FDA for osteoinduction [2,3]. BMP-2 and BMP-7 can also induce the differentiation of MSCs into osteoblasts and induce the mineralization of the extracellular matrix (ECM) produced by the osteoblasts. The exposure of mesenchymal stem cells (MSCs) to BMPs results in the sequential expression of osteoblast-specific markers [4]. BMP-2 activates the expression of the early marker transcription factor Runx2 (Runt-related transcription factor 2) [5]. This is followed by the activation of the gene of alkaline phosphatase (ALPL) and osteoblast-specific proteins, such as osteocalcin, osteopontin, and collagen type I. Furthermore, BMP-2 and BMP-7 induce the production of ECM components, such as glycosaminoglycans (GAGs), proteoglycans, and collagen type I, in MSCs [4]. The osteogenesis marker alkaline phosphatase (ALP) increased in a linear and non-linear manner with a growing level of BMP-7 and BMP-2, respectively [6]. Almodovar et al. showed that the ALP signal in the presence of both BMP-2 and BMP-7 was close to the sum of the signals when BMP-2 and BMP-7 were delivered separately on C2C12 myoblasts [6]. This suggests that BMP-2 and BMP-7 had an additive or synergistic effect [6,7].

Growth factors can be presented to biomaterial components via two main strategies, i.e., covalent binding and non-covalent binding [6]. Covalent binding involves the direct linking of the growth factor to the biomaterial [8], while non-covalent binding involves binding the growth factor to the biomaterial via its interactions with matrix proteins such as collagen, fibronectin, or laminin [9]. The layer-by-layer (LbL) technique of the deposition of polymers is an extremely versatile method for the bottom-up fabrication of functional coatings [10]. It allows for the application of different polycation–polyanion pairs, allowing for the easy tuning of the system chemistry and the attachment of growth factors. Additionally, this technique can be combined with microfluidic systems, allowing for the simultaneous investigation of multiple cellular signals (mechanical, biochemical, etc.) on the same substrate [11]. This combination of techniques offers a powerful and versatile platform for studying the behavior and interactions of cells in different environments. Natural polymers such as alginate (AG), cellulose, chitin, chitosan (CHI), dextrin, heparin (HP), hyaluronic acid (HA), and chondroitin sulfate (CS), and synthetic ones such as poly(glycolic acid) (PGA), poly(lactic acid) (PLA), polyurethanes, polyallylamine hydrochloride (PAH), etc., have been used extensively in the development of polymeric delivery systems of active substances [12,13,14,15]. These polymers are able to provide sustained drug release as well as increased stability and bioavailability. Additionally, the polymers can be used to prepare coatings, which can further improve the stability and effectiveness of the drug delivery system [16,17]. Glycosaminoglycans, i.e., HP, HA, CS, and heparan sulfate (HS), being the polyanionic components of the ECM, play a special role in developing new biomaterials [18]. Multilayer systems obtained from HP in combination with cationic chitosan (HP-CHI) hindered all inflammatory responses more significantly than analogous ones containing hyaluronic acid (HA-CHI) and chondroitin sulfate (CS-CHI) [19]. Malaeb et al. studied the binding of fibroblast growth factor (FGF-2) by biotinylated alginate sulfates and its influence on maintaining the growth of several neural/glial cell lines and regulating neurite outgrowth [20]. Wigmosta et al. showed an enhanced osteogenic effect of the BMP-2 after adsorption to CHI/gelatin multilayers on the titania surface [21]. Previously, we showed the effective immobilization of rhBMP-2 to diazoresin (DR, a synthetic polycation)–CS layouts [22]. rhBMP-2 was covalently and electrostatically bound to the negatively charged topmost layer. The immobilization of rhBMP-2 enhanced cell flattening while decreasing osteomarker expression. However, it should be mentioned that osteogenesis could be induced not only by selected growth factors, and the mentioned polysaccharides, but also by specific materials which enhance or stimulate the osteogenic cell response, such as hydroxyapatite, proteins (e.g., collagen and silk fibroin), synthetic polymers (e.g., poly(lactic acid) and poly(lactic-*co*-glycolic acid)), ceramics, or metals [23,24].

This paper presents the results of our studies on selected rhBMPs, namely rhBMP-2 and rhBMP-7, deposited on polymeric ultrathin multilayers composed of six DR/HP bilayers ((DR/HP)_6_), and on stem cells’ response to these systems. The rhBMP-2 and rhBMP-7, separately and as a combined system, were deposited on the outermost layer of the polymeric film, i.e., HP. The optimal conditions for in situ protein deposition studies were found using a quartz crystal microbalance with energy dissipation (QCM-D). The deposited proteins were covalently bonded via UV irradiation which also photocrosslinked the multilayer. Protein interactions with substrates were characterized using atomic force microscopy (AFM) and enzyme-linked immunosorbent assays (ELISAs). The response of human umbilical cord mesenchymal stem cells (hUC-MSCs), cultured on (DR/HP)_6_ in the absence and in the presence of rhBMP-2 and rhBMP-7 deposited separately or in combination, was investigated. The selected parameters of the cellular response were studied, including cytotoxicity, cell proliferation and flattening, cytoskeleton organization, migration into the artificial wound, and osteogenic marker expression. Importantly, the effect of various ionic strengths on both proteins deposited on DR/HP multilayer systems has not been described in the literature yet. The present research compares the cellular response to covalently bonded single proteins and a combination of both, using a versatile polymeric system based on HP.

## 2. Materials and Methods

### 2.1. Materials

Heparin sodium salt from porcine intestinal mucosa and 4-diazodiphenylamine sulfate were purchased from Sigma-Aldrich (USA). Sodium chloride, paraformaldehyde, zinc chloride, hydrogen peroxide 30%, sulfuric acid 96%, and isopropanol were received from POCh (Poland). Recombinant Human/Murine/Rat BMP-2 (CHO-derived) and recombinant human BMP-7 (CHO-derived) were purchased from Peprotech (USA). Mesenchymal stem cells in Wharton’s jelly of the human umbilical cord (hUC-MSC) were purchased from Promo Cell (Germany). Dulbecco’s Modified Eagle’s Medium/Nutrient Mixture F-12 Ham (DMEM/F12), heat-inactivated Bovine Serum Albumin (BSA), Triton X-100, and phosphate-buffered saline (PBS) were purchased from Sigma Aldrich (USA). Penicillin, streptomycin, Alamar Blue, and trypsin were purchased from Thermo Fisher Scientific (UK). Water was deionized using a Simplicity Millipore Water Purification System. All reagents were used as received. QCM sensors (14 mm in diameter, 5 MHz, Cr/Au/SiO_2_) were purchased from QuartzPro (Sweden). Silica wafers (11 mm × 11 mm) were received from Si-Mat (Germany). Glass coverslips (*Ø* = 15 mm) were bought from Mercateo (Poland). Culture flasks and plates were purchased from Sarstedt (Germany).

### 2.2. Multilayer Preparation and Protein Deposition

Paraformaldehyde and 4-diazodiphenylamine were used as reactants for diazonium resin (DR) synthesis. The synthesis pathway and procedure have been reported previously [25]. The layers were deposited from 2 mg/mL DR solutions in deionized water and 1 mg/mL HP solution in 0.1 M NaCl. As previously reported, the multilayer polymeric films were prepared on glass coverslips (clean substrate) [22]. Multilayers composed of six DR/HP bilayers ((DR/HP)_6_) with the anionic HP as a top layer were prepared. The photocrosslinking of the DR/HP films with or without photoimmobilized BMPs on their surfaces was carried out using a UV lamp with the maximum emission intensity at 350 nm. The covalent bond formation between DR and HP is shown schematically in Figure S1.

To monitor the in situ deposition of rhBMP-2 and rhBMP-7 onto sensors coated with the (DR/HP)_6_, a quartz crystal microbalance with a dissipation monitoring system (QCM-D, Q-Sense, Gothenburg, Sweden) was used. The ionic strength of protein solutions during deposition was optimized. Sensor surfaces were washed with isopropanol and dried under the inert gas flow, and polymers were deposited according to the procedure described previously [22] and measured according to the standard method described in the literature [26]. A stable baseline for water or appropriate NaCl solution was obtained at various ionic strengths (1.0 × 10^−3^, 1.0 × 10^−2^, 0.15 M). After the stabilization of the baseline, an rhBMP-2 solution or rhBMP-7 (1 μg/mL) was pumped through the cell at a flow rate of 0.15 mL/min for 30 min. Subsequently, pure water or appropriate NaCl solution was flushed through the cell to study the protein molecule desorption. The mass of adsorbed rhBMP-2 or rhBMP-7 per unit area (coverage) was calculated using Sauerbrey’s equation (for details, see [27]).

**Figure 1:**
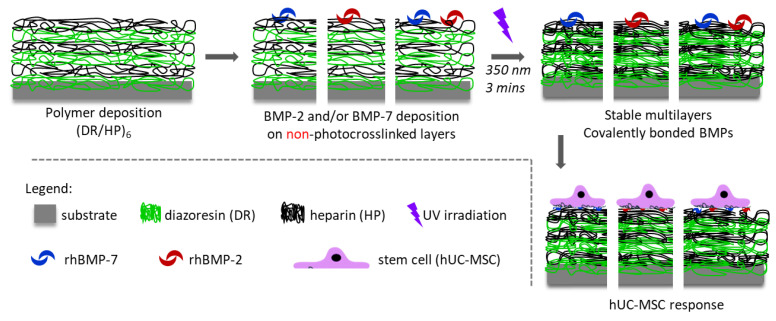
Schematic presentation of the preparation of different substrates.

RhBMP-2 and rhBMP-7 were deposited onto the polymeric surface with the HP as a top. The proteins were deposited separately before the UV irradiation of the multilayers. The substrates were incubated in 1 µg/mL of rhBMP-2 or rhBMP-7 aqueous solutions (incubation conditions: 15 min and air saturated with water vapor). Then, the substrates were gently washed and photocrosslinked for 3 min to covalently bind the proteins to the surface. Four systems were finally obtained and examined: (DR/HP)_6_; (DR/HP)_6__BMP-2; (DR/HP)_6__BMP-7; and (DR/HP)_6__BMP-2/-7 (Figure 1).

### 2.3. Surface Analyses

An atomic force microscope (AFM) (Bruker Dimension ICON XR, Germany) working in tapping mode was used to characterize the surfaces with and without immobilized proteins in the dry state. Standard silicon cantilevers (Bruker) with a nominal spring constant equal to 0.4 N/m were used for all of the measurements. Images were analyzed using the dedicated NanoScope Analysis software.

Polymeric substrates with rhBMP-2 and rhBMP-7 adsorbed on the surface were incubated at 37 °C in a 5% CO_2_ atmosphere. At selected time points (3, 7, 10, and 14 days), aqueous solutions of both proteins released from the substrates were collected and stored at −80 °C until analysis. At each step, the new portion of 1.0 mL of fresh 1.0 × 10^−2^ M solution of NaCl was added to each well for the releasing test of rhBMP-2 and rhBMP-7. The concentrations of proteins were measured by using commercial competitive ELISA kits (intra-assay CV < 8%, inter-assay CV < 10%, Mouse BMP-2 Elisa Kit and Human BMP-7 Elisa Kit, ELISA Genie, UK). An ELISA microplate reader Infinite M Nano (Tecan, Mannedorf, Switzerland) was used to determine the absorbance of the samples at 450 nm. The experiments were carried out in triplicate.

### 2.4. Cell Culture

Primary human umbilical cord mesenchymal stem cells (hUC-MSCs) were bought from PromoCell. The purchased MSCs were isolated, characterized, and confirmed as being multipotent by the manufacturer. hUC-MSC was cultured in a DMEM/F12 supplemented with 2% or 10% FBS and 100 IU/mL penicillin and 10 μg/mL streptomycin at 37 °C in a humidified atmosphere with 5% CO_2_ in all in vitro experiments. All cellular experiments were performed on passages between 3-5.

### 2.5. Cell Viability and Proliferation

Glass coverslips covered by the (DR/HP)_6_ films were placed in a 24-well culture plate. RhBMP-2 and rhBMP-7 were deposited under selected conditions and photocrosslinked. The cytotoxic effect of the composed systems was analyzed using the Alamar Blue assay. hUC-MSCs were seeded in 1.5 × 10^4^ cells/well density in DMEM/F12 medium with 10% FBS. After 24 h, the culture medium was removed and cells were washed with PBS. The culture medium was mixed with 10% vol. of the Alamar Blue and added to each well. Cells were incubated for 2 h in an incubator at 37 °C. After that time, 200 μL aliquots of the mixture were collected from each well and placed into a 96-well plate, and the absorbance at λ = 570 nm (ref. 600 nm) using a plate reader (Infinite M Nano, Tecan, Switzerland) was read. The next time points were 48 h, 72 h, and 7 days. The (DR/HP)_6_ system was used as a control.

Cell proliferation was analyzed using the hemocytometer and dye exclusion test (Trypan Blue Solution, 0.4%, Thermo Fisher Scientific, Waltham, MA, USA). Glass coverslips covered by the (DR/HP)_6_ films were placed in a 24-well culture plate. rhBMP-2 and rhBMP-7 were deposited under selected conditions and photocrosslinked. hUC-MSCs were seeded at 1.5 × 10^4^ cells/well density in DMEM/F12 medium with 10% FBS. At the selected time points, i.e., 2, 3, and 7 days, cells were washed 2-times with PBS, detached using 100 μL of trypsin/well washed with 500 μL of culture medium, centrifuged, and resuspended in 1 mL of culture medium. A total of 10 μL of cell suspension was mixed with 10 μL of Trypan Blue solution to stain the dead cells. Only non-stained cells were counted using the hemocytometer under an inverted optical microscope (Axio Vert.A1, Zeiss, Dresden, Germany). The (DR/HP)_6_ system was used as a control.

### 2.6. Cytoskeleton Organization and Cell Flattening

hUC-MSCs were seeded on coverslips coated with a (DR/HP)_6_ multilayer without proteins (control) and with proteins, at a density of 1.5 × 10^4^ cells/well in a 12-well culture plate. The cells were cultured in DMEM/F12 medium with 10% FBS for 24 h. Next, they were fixed using the standard protocol in warm 3.7% formaldehyde for 15 min, solubilized with 0.1% Triton X-100 in PBS for 7 min, and washed in PBS. Then, cells were immunostained with mouse monoclonal anti-human vinculin IgG in PBS solution of 3% BSA (Merck, Darmstadt, Germany). Alexa Fluor-488-conjugated goat anti-mouse IgG-clone A11001 (Merck, Darmstadt, Germany) was counterstained with TRITC-phalloidin (Merck, Darmstadt, Germany), according to the manufacturer’s protocol. Nuclei were stained with DAPI following the manufacturer’s protocol (Merck, Darmstadt, Germany). The specimens were mounted onto coverslips with poly(vinyl alcohol) (Dako Fluorescent Mounting Medium, Agilent Technologies, Santa Clara, CA, USA). An inverted fluorescence microscope (Axio Vert.A1, Zeiss, Dresden, Germany) was used to visualize the cells which were analyzed using ZEN 2.3 (Zeiss, Dresden, Germany) software.

### 2.7. Cell Migration

Glass coverslips covered by the (DR/HP)_6_ films were placed in a 24-well culture plate. rhBMP-2 and rhBMP-7 were deposited under selected conditions and photocrosslinked. hUC-MSCs were seeded on coverslips coated with a (DR/HP)_6_ multilayer without proteins (control) and with proteins, at a density of 14 × 10^4^ cells/well, and incubated in a medium supplemented with 2% FBS. After 24 h, cells formed a monolayer, and the medium was replaced with a fresh one. Analyses were performed using the cells moving into the wound experimental model, as described previously [28]. A cell-free area was introduced by scraping the monolayer using a 100 μL pipette tip and then cell migration into the cell-free area was controlled under an inverted microscope (Axio Vert.A1, Zeiss, Dresden, Germany). The overgrowth of the wound was monitored by taking pictures of a given area at different time points (0, 2, 4, 8, and 12 h), calculating the percentage of the disappearance of the wound surface area. The results are relative to the control, i.e., slides with cells grown in medium with 2% FBS.

### 2.8. Osteogenic Differentiation

Glass coverslips covered by the (DR/HP)_6_ films were placed in a 24-well culture plate. rhBMP-2 and rhBMP-7 were deposited under selected conditions and photocrosslinked. hUC-MSCs were seeded on clean glass coverslips coated with a (DR/HP)_6_ multilayer without proteins (control) and with proteins ((DR/HP)_6__BMP-2; (DR/HP)_6__BMP-7; and (DR/HP)_6__BMP-2/-7), at a density of 1.5 × 10^4^ cells/well, and incubated in a medium supplemented with 10% FBS. After 6 h, when cells adhered to all culture surfaces, the medium was changed to the fresh one supplemented with 2% FBS. The medium was exchanged every 3 days. After 7 days, the total cellular RNA was isolated (Gene MATRIX Universal RNA Purification Kit, Eurx Ltd., Gdansk, Poland) following the manufacturer’s protocol. Next, reverse transcription was performed using 1 µg RNA, an NG dART RT-PCR kit (Eurx Ltd., Gdansk, Poland), and the C1000 Touch Thermal Cycler (Bio-Rad Laboratories, Hercules, CA, USA). qRT-PCR analysis was employed using the QuantStudio 6 Flex Real-Time PCR System (Thermo Fisher, Waltman, MA, USA), SYBR Green Master Mix (Eurx Ltd., Gdansk, Poland), 100 ng cDNA, and 7.5 µM of specific primer to detect selected genes activated during osteogenesis, such as the following: alkaline phosphatase (ALPL), Runt-related transcription factor 2 (RunX2), and osteocalcin (OCN) (Merck, Darmstadt, Germany) genes. The sequences of the utilized primers are included in Table S1 (see the Supplementary Materials). The mRNA expression level was normalized to the housekeeping gene GAPDH. The qRT-PCR conditions were as follows: 95 °C for 15 min, 40 cycles of denaturation (15 s, 94 °C), annealing (30 s, 55 °C), and extension (30 s, 72 °C). The 2(−^ΔΔ^)Ct method was employed to determine the relative mRNA expression in different samples, by calculating the fold change in expression within the different cultured cells with respect to the control group ((DR/HP)_6_) [29]. The mRNA expression in the control was considered to be 1 in all experiments, and the expression (normalized to the GAPDH gene) in the experimental group was calculated as a fold of the expression level in the control groups.

### 2.9. Data Analysis

Statistical analysis was performed using GraphPad Prism software (GraphPad Software, San Diego, CA, USA). One-way ANOVA and Bonferroni (post hoc test) tests were applied. p values less than 0.05 (*p* < 0.05) were considered to be statistically significant. Statistically significant differences were labeled with an asterisk (*).

## 3. Results

### 3.1. Polymeric Substrate Preparation

The procedure of the substrate preparation is shown in Figure 1. The multilayers composed of DR and HP were deposited on clean glass coverslips via the alternate immersion of the substrate in DR and HP solutions. The synthetic cationic DR and natural anionic HP were deposited alternately, ending the outermost layer with HP. The polymeric system was composed of six bilayers (DR/HP)_6_, starting from DR and ending with HP.

### 3.2. Protein Deposition and Substrate Characterization

Human recombinant bone morphogenetic protein-2 (rhBMP-2) and human recombinant bone morphogenetic protein-7 (rhBMP-7) were deposited separately and in combination on non-photocrosslinked multilayers. Quartz crystal microbalance with dissipation monitoring (QCM-D) was used to analyze the efficiency of rhBMP-2 and rhBMP-7 deposition on Au/SiO_2_ sensors coated with the (DR/HP)_6_ multilayer. Aqueous solutions of NaCl in the volumes of 1.0 × 10^−3^, 1.0 × 10^−2^, and 0.15 M and pure water were used to suspend the proteins and check the amounts of proteins that were adsorbed on the surface. Table 1 shows the mean mass (calculated from the frequency overtones) of rhBMP-2 and rhBMP-7 that was adsorbed on HP within 30 min of protein flow. The main aim was to deposit the highest amount of both proteins using the same solvent.

**Figure 2:**
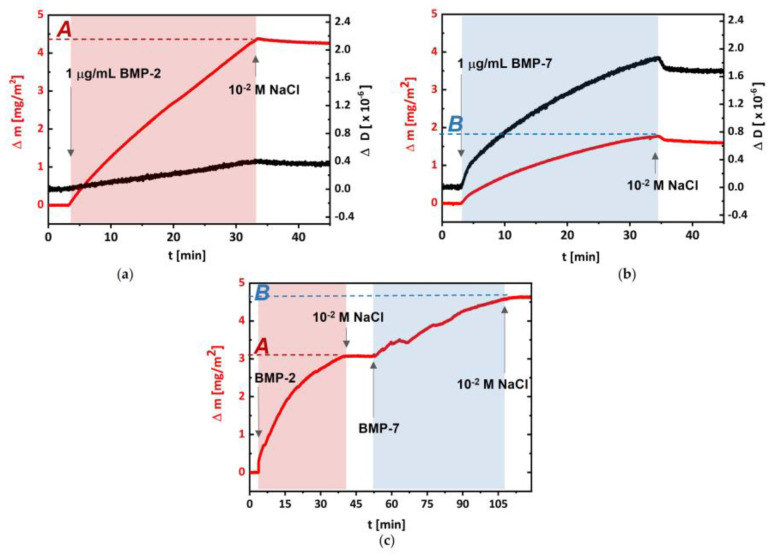
(**a**) rhBMP-2, (**b**) rhBMP-7, and (**c**) rhBMP-2/rhBMP-7 adsorption/desorption run for the Au/SiO_2_ sensor coated with non-photocrosslinked (DR/HP)_6_, expressed as the mass shift (red curves) and dissipation shift (black curves). rhBMPs were deposited from 1 μg/mL solution in 1.0 × 10^−2^ M NaCl at pH 6.2 and a flow rate of 0.15 mL/min. rhBMPs were deposited for about 30–60 min (marked as light red or light blue areas) and points A and B represent the adsorbed protein mass.

The deposition of proteins separately (Figure 2a,b), as well as in a sequence (rhBMP-2/rhBMP-7), on the same substrate (Figure 2c) was performed under optimized conditions, i.e., protein concentration 1 μg/mL, pH 6.2, and ionic strength I = 1.0 × 10^−2^. The process of rhBMP-2 adsorption on (DR/HP)_6_ led to Δm = 4.4 μg/cm^2^ and ΔD = 0.4 × 10^−6^ (Figure 2a), whereas the adsorption of rhBMP-7 to Δm = 1.85 μg/cm^2^ and ΔD = 1.7 × 10^−6^ (Figure 2b). The rinsing step did not significantly remove the adsorbed proteins in both cases; however, a little more of rhBMP-7 was detached during this step. To better control the adsorption of selected proteins, they were not deposited simultaneously (i.e., from the mixture solution) but one after another as the third system (Figure 2c). Starting with the adsorption of rhBMP-2, the Δm = 3.1 μg/cm^2^ coverage was obtained, and was followed with rhBMP-7 adsorption, which led to the deposition of Δm = 1.5 μg/cm^2^, confirming the formation of the system with both proteins adsorbed, denoted as (DR/HP)_6__BMP-2/-7. The order of protein deposition was chosen based on the slightly greater desorption of rhBMP-7 compared to rhBMP-2. In all cases, after protein deposition, they were bound to the multilayer via UV irradiation which induced the photochemical formation of covalent bonds between protein molecules and the polymeric multilayer.

**Figure 3:**
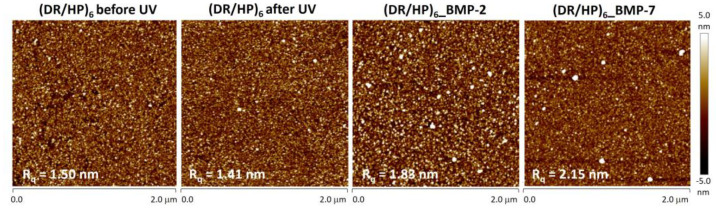
Representative topography images of (DR/HP)_6_ multilayers immersed in 1 × 10^−2^ M NaCl before and after photocrosslinking and after the deposition of rhBMP-2 and rhBMP-7 from 1.0 µg/mL solutions. Measurements were performed at room temperature using the tapping mode and were collected from two areas. The corresponding root mean square roughness (*R*_q_) values are shown in each image.

We used atomic force microscopy to analyze the topography of the (DR/HP)_6_ systems obtained. Figure 3 (two left images) shows the topography of the (DR/HP)_6_ layout before and after UV irradiation. It indicates that the photocrosslinking caused a slight decrease in surface roughness, *R*_q_, from 1.50 nm to 1.41 nm. This result is in good agreement with the literature reports [22]. Proteins were deposited from 1 μg/mL solutions with ionic strength *I* = 1.0 × 10^−2^. Figure 3 (two right images) shows the surface topography of the (DR/HP)_6_ system with covalently bound proteins (after photocrosslinking), whose molecules are marked as white spots. Surface decoration with proteins increased surface roughness to 1.83 nm and 2.15 nm for rhBMP-2 and rhBMP-7, respectively.

Using the enzyme-linked immunosorbent assay (ELISA), it was verified as to whether the proteins were released from the surfaces in spite of their covalent binding to the surface. The release profiles are presented in Figure S2. The release of both proteins was analyzed on Days 3, 7, 10, and 14. As can be seen, only rhBMP-2 was released at 48% until the 3rd day of incubation. Until Day 7, the rest of this protein was released. rhBMP-7 did not desorb into the solution within two weeks of the experiment.

### 3.3. Substrate Cytotoxicity

**Figure 4:**
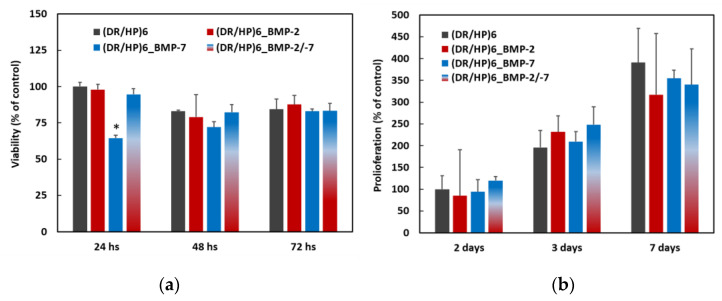
(**a**) Cell viability evaluated after 24, 48, and 72 h of hUC-MSC culture on (DR/HP)_6_—control, (DR/HP)_6__BMP-2, (DR/HP)_6__BMP-7, and (DR/HP)_6__BMP-2/-7. (**b**) Proliferation evaluated after 2, 3, and 7 days of hUC-MSC culture on (DR/HP)_6_—control, (DR/HP)_6__BMP-2, (DR/HP)_6__BMP-7, and (DR/HP)_6__BMP-2/-7. The results with p values less than 0.05 (*p* < 0.05) were considered to be statistically significant in comparison to the control and were labeled with an asterisk (*).

Cell viability and proliferation are crucial for the potential biomedical application of the material. We analyzed the effect of the composed substrates on the viability of hUC-MSCs. The cell viability was analyzed at 3 time points: 24, 48, and 72 h, using the Alamar Blue assay (Figure 4a) [30]. All results were normalized to the control (DR/HP)_6_ system at the appropriate time point. As can be seen, all systems, except (DR/HP)_6__BMP-7, showed similar cellular viability, compared to the control. The viability of cells cultured on (DR/HP)_6__BMP-7 slightly decreased compared to the control, but within 48 and 72 h of cell culture, the cells appeared to adapt to the new surface conditions, and with the extension of the culture time, the differences in cell viability among the substrates decreased. The cell proliferation was analyzed after 2, 3, and 7 days (Figure 4b) of cell culture via Trypan Blue exclusion counting in the hemocytometer [31]. After 2 days of the cell culture, the number of cells was significantly higher for all of the composed systems. Between Day 2 and 3, the number of cells cultured on the control and on (DR/HP)_6__BMP-2/-7 doubled, while the number of cells increased around 2.7 and 2.2 times for (DR/HP)_6__BMP-2 and (DR/HP)_6__BMP-7, respectively. After 7 days of cell culture, the number of cells was similar for all of the conditions.

### 3.4. Cell Flattening and Migration

**Figure 5:**
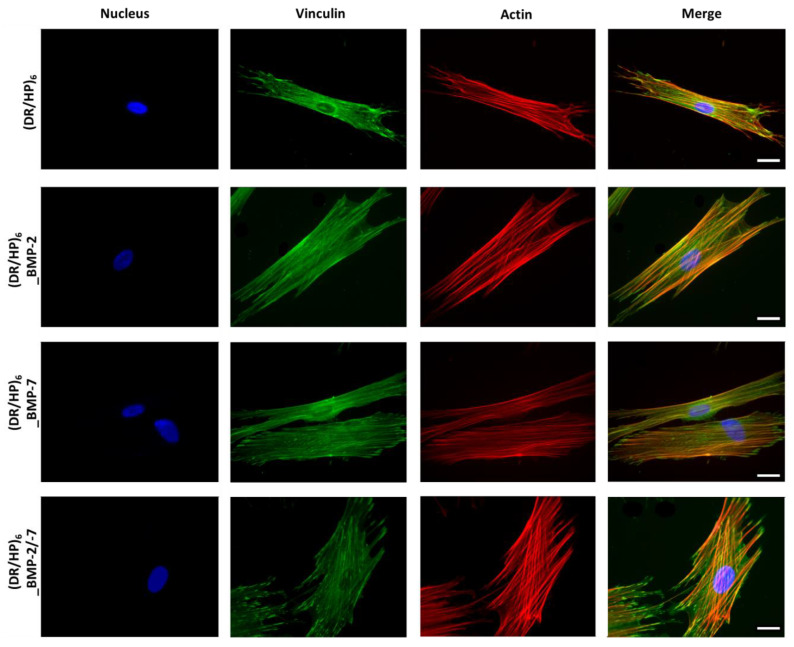
Actin cytoskeleton architecture of hUC-MSCs. Cells were cultured for 24 h on (rows from top to bottom) (DR/HP)_6_ polymeric system without proteins (control) and with immobilized rhBMP-2 or rhBMP-7 and both proteins combined, in a medium supplemented with 2% FBS. Cells were immunostained with vinculin and counterstained with TRITC-phalloidin (F-actin) and DAPI (nuclei). The scale bar represents 25 µm.

Cell adhesion is the first reaction of cells to the substrate of a biomaterial. hUC-MSCs were seeded on composed substrates, and their morphology and cytoskeleton organization were observed during the 1st contact with the material (24 h after seeding). Cells were immunofluorescently stained with vinculin and counterstained with TRITC-phalloidin for F-actin (Figure 5). The nuclei were fluorescently stained using DAPI. Figure 5 shows the significant difference in cell morphology and cytoskeleton organization in each condition. For the (DR/HP)_6_ (control), (DR/HP)_6__BMP-2, and (DR/HP)_6__BMP-7 substrates, the hUC-MSCs assumed a more elongated shape than the substrate with two deposited proteins. Cells cultured in all three conditions with deposited proteins were more flattened than those cultured on (DR/HP)_6_. Microfilament bundles (stress fibers, red) were well organized along the cells for these conditions. Actin filaments were well strained. Furthermore, significant morphological changes could be observed for cells cultured on the (DR/HP)_6__BMP-2/-7 system. Cells assumed a more oval shape and were more flattened than for the single protein systems, which induced a more elongated shape of the cells. Moreover, cells on the (DR/HP)_6__BMP-2/-7 system were anchored to the substrate via the creation of vinculin-rich focal adhesion complexes at the end of the actin filaments. Cells cultured in single protein systems formed more microspikes and pseudopodia due to their attempt to move.

**Figure 6:**
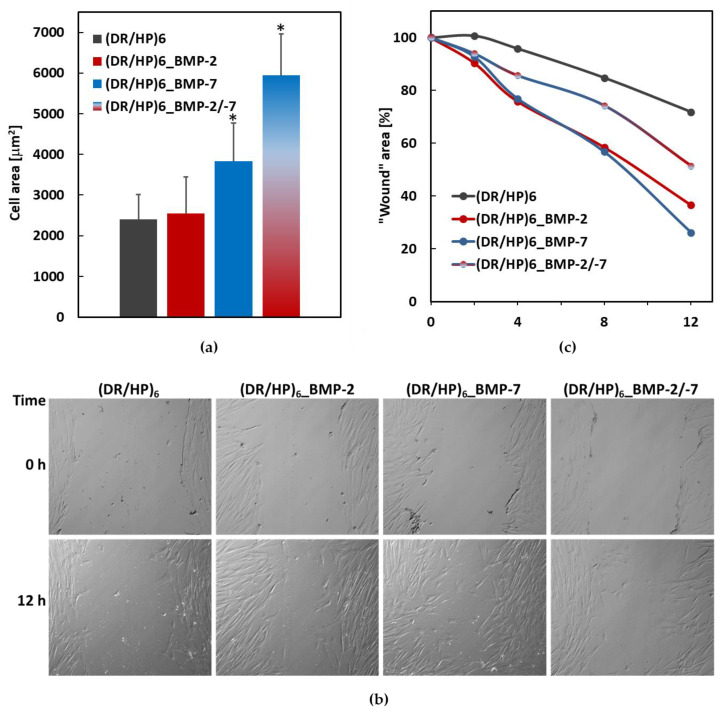
(**a**) Cell area 24 h after seeding measured for 20 cells cultured on various surfaces. Results with *p* values less than 0.05 (*p* < 0.05) were considered to be significantly different from the control and were labeled with an asterisk (*). (**b**) hUC-MSC migration into the “wound”. Cells cultured on (DR/HP)_6_, (DR/HP)_6__BMP-2, (DR/HP)_6__BMP-7, and (DR/HP)_6__BMP-2/-7 were monitored at the time points of 0, 2, 4, 8, and 12 h. (**c**) Micrographs of hUC-MSC migrating into the scratched cell-free areas taken immediately after “the injury” and after 12 h of continuous cell migration into the wound.

The mean cell area was calculated for 20 cells cultured on various surfaces (Figure 6a). For the systems (DR/HP)_6_ and (DR/HP)_6__BMP-2, the area was about 2.5-fold larger (~2400 µm^2^ and ~2500 µm^2^, respectively). The area of hUC-MSC cultured on the (DR/HP)_6__BMP-7 system was 1.5-fold larger than that of the cells cultured on (DR/HP)_6__BMP-2. The cells assumed the most flattened form on (DR/HP)_6__BMP-2/-7 with the area of ~6000 µm^2^. This value was statistically significant compared to the other conditions.

Next, we evaluated the hUC-MSC migration into a model wound mechanically generated within a cell monolayer seeded on the composed polymeric substrates [28]. Cells were cultured in the culture medium supplemented with 2% FBS in order to limit their proliferation. Wound images were taken immediately after the scratch was made, after 2, 4, 8, and 12 h of continuous cell culture (Figure S3). Figure 6b shows the micrographs of hUC-MSCs migrating into the scratched cell-free areas taken just after the injury (t = 0 h) and after 12 h. The results are expressed as the % of wound closure (Figure 6c). As can be seen, cell migration was the slowest in the (DR/HP)_6_ system (black line). Within 12 h, the wound area decreased by only 30%. For the systems decorated with single proteins, the wound was overgrown by about 8% after 2 h, 24% after 4 h, and 42% after 8 h. After 12 h, the wound area decreased to 36% and 26% for rhBMP-2 and rhBMP-7, respectively. Cells cultured on (DR/HP)_6__BMP-2/-7 migrated almost two times slower compared to single protein systems, but at the same time migrated two times faster than cells cultured on (DR/HP)_6_.

### 3.5. Osteomarker Expression

**Figure 7:**
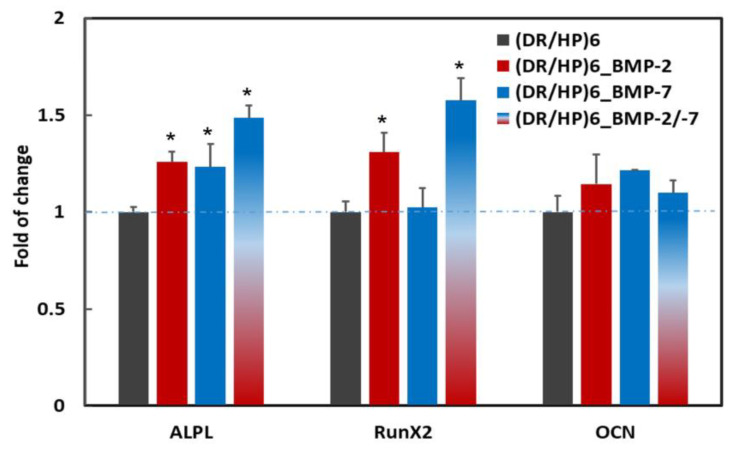
The expression of selected osteogenic gene markers in hUC-MSC cultured for 7 days on (DR/HP)_6_—control, (DR/HP)_6__BMP-2, (DR/HP)_6__BMP-7, and (DR/HP)_6__BMP-2/-7. Quantitative real-time PCR analysis of gene expression of *ALPL*—alkaline phosphatase; *RunX2*—Runt-related transcription factor 2; and *OCN*—osteocalcin. Gene expression was normalized to the expression of the housekeeping gene *GAPDH*—glyceraldehyde-3-phosphate dehydrogenase—and shown as the fold change compared to the gene expression of hUC-MSC cultured on the (DR/HP)_6_ surface (blue line). Results with *p* values less than 0.05 (*p* < 0.05) were considered to be significantly different in comparison to the control and are labeled with an asterisk (*).

After 7 days of hUC-MSC culture on different types of polymeric surfaces, the gene expression level of selected osteogenic markers was analyzed (Figure 7). To demonstrate the proteins’ effect on hUC-MSC differentiation, the gene expression was presented relative to the expression of a single gene in hUC-MSC cultured on the (DR/HP)_6_ surface. The statistically significant effect of polymeric surfaces on hUC-MSCs was observed in the expression of the *ALPL* gene (the early osteogenic marker). The expression level of *ALPL* in the single protein systems (DR/HP)_6__BMP-2 and (DR/HP)_6__BMP-7) increased by approximately 25%, while for the double protein system ((DR/HP)_6__BMP-2/-7) it increased by 50% compared to the control. The gene expression analysis of another early osteogenic marker—*RunX2*—indicated a significant increase for (DR/HP)_6__BMP-2 and (DR/HP)_6__BMP-2/-7 equal to 30 and 60%, respectively, while for (DR/HP)_6__BMP-7 the expression of *RunX2* did not change compared to the control. Moreover, a slight, although not statistically significant, increase in the expression of *OCN* (the middle osteogenesis marker) in hUC-MSCs for all of the analyzed systems was observed. In particular, the (DR/HP)_6__BMP-7 polymer demonstrated a 20% increase in *OCN* expression in comparison to the control.

## 4. Discussion

Combined diazoresin–polyanion systems can act as versatile ultrathin platforms for stable covalent bond formation [32]. In this study, synthetic cationic DR and natural anionic HP were deposited alternately making a six-bilayer system, ending with the outermost layer with HP to make it more biocompatible. During irradiation with 350 nm light, the covalent bonds between the layers and between the top layers and the studied growth factors were formed, accompanied with the release of the gaseous nitrogen and formation of phenyl cations in the DR structure. In our system, the phenyl cations reacted with sulfate or the carboxyl groups of HP and the carboxyl groups of the proteins, forming C-S and C-O covalent bonds, respectively (Figure S1). The bond formation can be confirmed via the UV-Vis absorption spectra (decrease in the intensity of the diazonium group absorption band; data not shown). The detailed analysis of the DR-HP layer interactions was shown in our previous paper [33]. The non-photocrosslinked HP-terminated system had a surface charge of around -70 mV, while after irradiation, it increased to -50 mV [33]. We have shown that rhBMP-2 can be deposited on similar polymeric films using CS as a polyanion instead of HP [22]. However, rhBMP-2 was effectively bonded to the negatively charged terminal layer only, no matter whether it was cross-linked or not. The presented research focused on the deposition of two osteoinductive homodimeric proteins: rhBMP-2 and rhBMP-7. Proteins were deposited individually on the (DR/HP)_6_ multilayer, forming (DR/HP)_6__BMP-2 and (DR/HP)_6__BMP-7, and one after another on the same substrate, forming a (DR/HP)_6__BMP-2/-7 system.

The QCM-D technique measures two quantifiable parameters, the resonance frequency shift (related to the detected mass coverage *Δm* of the surface-bound protein) and the energy dissipation shift (related to the viscoelastic properties of the adsorbed layer *ΔD*) [22]. In order to deposit both proteins using the same experimental conditions, we had to find the optimal ionic strength. In the first step, the adsorption of single proteins on the (DR/HP)_6_ multilayer, as a function of ionic strength, was optimized via monitoring using QCM-D measurements and deposited mass analysis. In the case of the rhBMP-2 interplay with HP, there are literature reports indicating that it possesses a specific HP-binding site [34]. Furthermore, Kanzaki et al. investigated the affinity between HP and rhBMP-2 using a QCM technique and confirmed that HP competitively inhibits the binding of BMP-2 and BMPR [35]. On the other hand, HP can act as a vector for rhBMP-2 and can be used to improve bone formation [36]. The larger the ionic strength is, the higher the amount of rhBMP-7 that can be deposited due to the salting out process. According to the obtained data, the optimal ionic strength for the adsorption of both proteins on the (DR/HP)_6_ multilayer was *I* = 1.0 × 10^−2^ (Table 1). This ionic strength enables the deposition of both protein layers with higher coverage (rhBMP-2 *Δm* = 4.4 μg/cm^2^; rhBMP-7 *Δm* = 1.85 μg/cm^2^) than at lower *I* (Figure 3). These data imply that the rhBMP-2 protein formed a layer with higher coverage and a tighter (more rigid) structure on the (DR/HP)_6_ multilayer in comparison to rhBMP-7 in the same experimental conditions (ionic strength, protein concentration, type of substrate, flow rate, and adsorption time). As mentioned above, the phenomenon can be explained by stronger interactions of rhBMP-2 with HP. Although the details of the mechanism of action of the HP fragments remain to be elucidated, it has been determined that HP prolongs BMP-2-induced cell signaling, as confirmed by the phosphorylation of Smad 1/5/9 [37]. Smith et al. indicated a minimum requirement chain length of ten monosaccharides dp10, optimally a dp12, and maximally a dp16 for the binding of HP to BMP-2 [38]. Furthermore, they confirmed the BMP-2 preference for the binding to the *N*-sulfated HP/HS domains of dp10 or greater. A previous study demonstrated that rat MSCs cultured with de-2-*O*-sulfated HP and BMP-2 revealed enhanced MSC proliferation and ALP activity in comparison to native HP [39]. To avoid any undesired protein–protein interactions, we decided to deposit rhBMP-2 and rhBMP-7 separately onto one substrate, i.e., one after another. Figure 2c shows the *Δm* changes for the deposition of both proteins. In both cases, the mass of rhBMP-2 deposited was twice that of rhBMP-7. The AFM measurements showed that the protein amount correlated well with the QCM-D measurements. As can be seen, a higher amount of rhBMP-2 was adsorbed on the surface (white spots) than that of rhBMP-7 (Figure 3). The presence of the protein increased the surface roughness. It should be emphasized that the QCM for the protein deposition technique as a dynamic system gives the amount of proteins as being higher than that found using the static deposition model.

The rhBMP-2 and rhBMP-7 release profiles were analyzed using the enzyme-linked immunosorbent assay (ELISA) with the selected time points of 3, 7, 10, and 14 days. This was the standard protocol of medium culture exchange, which was conducted twice per week. According to the assay manufacturer, it detects both proteins only in the biologically active free form. As can be seen in Figure S2, rhBMP-2 was released from the substrate for up to 7 days. The literature shows that neither photoimmobilization nor the formation of the covalent bond caused rhBMP-2 denaturation [40,41]. rhBMP-7 was not detected in the milieu. Nevertheless, most probably, both proteins are still stably bound to the surface as suggested by the different cellular responses on the composed layouts compared to the single protein layouts and the control.

Cell viability and proliferation on the surface of a biomaterial are important for successful material–tissue biointegration. Cells need to be able to adhere to the surface to proliferate on it [42,43]. Factors such as surface roughness, composition, and chemistry can all affect cell viability and proliferation on the surface of a material. The first contact of cells with the biomaterial is crucial in the context of their further behavior around the implant. According to our results, composed systems do not significantly affect cell viability within short-term interactions (up to 24 h). The only one condition which decreased cell viability was by depositing BMP-7 within 24 h. The short-term interactions of the biomaterial with cells are important for further stages of material integration. However, cell viability was at the same level in the following days, regardless of the substrate type. The same tendency was observed for the proliferation assay. After 48 and 72 h, the cell number did not increase significantly compared to 24 h of culture. Furthermore, the cell number became similar for both systems, indicating that the effect of the initial interaction diminished after a couple of days (short-term cultivation).

More noteworthy changes were observed for cell flattening and migration. The images showed a significant difference in cell morphology and cytoskeleton organization at the beginning of the cell–material interactions (up to 24 h) (Figure 5). It is well established that protein adsorption can significantly change the local microenvironment of a material. This is because the adsorbed proteins, not only as active molecules, can alter the surface chemistry, topography, and charge of the material, influencing selected signaling pathways induced in cells [44,45]. All systems decorated with proteins significantly affected cell morphology. They were more elongated while cultured on single proteins, whereas they assumed an oval, strained shape on both deposited proteins, which corresponds well with cell areas (Figure 6a). hUC-MSC cultured on surfaces with rhBMP-2 and rhBMP-7 immobilized formed, dense, and prominent microfilament bundles (actin stress fibers, shown in red) and vinculin-rich focal adhesion complexes [46]. Although adhesion is a complex interaction process, the crucial role is the interplay between integrins and BMP receptors [47]. Recent studies have shown that rhBMP-7 directly upregulates the adhesion and migration of human monocytic cells via the activation of β2 integrins, Akt, and FAK [48]. rhBMP-2 on Ti discs also improved C2C12 cell adhesion and spreading by increasing the vinculin expression [49]. Laflamme et al. demonstrated that the mixture of rhBMP-2 and rhBMP-7 was more effective than the separately acting homodimers in promoting osteoblast adhesion and proliferation [50]. Our results support previously published reports indicating that different BMPs can act additively.

Cell adhesion is closely linked to cell migration ability. Integrins are the main proteins involved in cell adhesion, and they interact with components of the ECM to form strong bonds between the cell and the substrate [51]. Cell adhesion is also essential in regulating cell migration speed and direction. According to our studies, the presence of both proteins results in the lowest migration ability of hUC-MSCs, compared to single proteins. The inhibition of hUC-MSC migration on (DR/HP)_6__BMP-2/-7 is a consequence of strong cellular adhesion and flattening caused by cell–material interplay. In the case of separately deposited rhBMP-2 and rhBMP-7, the cell shape is much more elongated, which could enhance the migration into the artificial wound (Figure 6b). A different result was observed in the case of pure polymeric film. Namely, the cells were not significantly flattened and exhibited the lowest migration activity. It can be concluded that very active rhBMPs outperform the pure polymeric substrate in activating hUC-MSC target signaling pathways. Our polymeric platform can act as a versatile substrate for protein deposition. The inhibition of cell migration is a desired effect in the first cell–biomaterial contact immediately after implant placement.

Finally, we analyzed the effect of composed systems on the expression of markers of different osteogenesis phases. An interesting correlation was observed for proteins’ deposited layouts with early osteogenesis markers, i.e., *ALPL* and *RunX2* gene expression. For the (DR/HP)_6__BMP-2/-7 system, rhBMP-2 and rhBMP-7 acted additively with respect to the *ALPL* marker, while the synergistic effect was observed for the *RunX2* marker (Figure 7). This correlates well with the literature reports about the additive interplay of these proteins [50]. Based on the obtained results, we can conclude that deposited proteins still exhibit osteoactivity, which is increased by the deposition of both proteins combined. A single rhBMP-2 or rhBMP-7 deposition increased the expression of these early markers, but to a lesser extent than the deposition of combined proteins. These results are crucial from a cell differentiation point of view and for cell adhesion on the implant surfaces. It was confirmed that BMP-2 activates the expression of *RunX2* and transcription factor (*Sp7*), obligatory transcription factors for osteoblast differentiation, and induces osteoblastic markers such as *ALP*, *COL1A1*, and osteocalcin (*OCN*) [52]. BMP-7 stimulates the mineralization of the extracellular matrix and the activity of ALP [53]. Brigaud et al. showed the synergistic potential of the BMP-2 and BMP-7 deposition on Ti-hydroxyapatite-fibronectin (Ti-HA-FN). They established that a minimum of ~2.0 μg/cm^2^ of adsorbed BMP-2 or 1.1 μg/cm^2^ of adsorbed BMP-7 was necessary to trigger the osteogenic activity of C2C12 cells [2]. Furthermore, when C2C12 cells were cultured on these BMP-2 biomimetic (PLL/HA) films, significant *ALP* activity was detected from 1.4 μg/cm^2^ for BMP-2 and 1.0 μg/cm^2^ for BMP-7 [2]. They also showed that FN interplay with BMP-7 did not increase the *RunX2* marker, while FN combined with BMP-2 and BMP-7 separately increased the level of the *ALPL* marker. This agrees with our studies, which showed that rhBMP-7 deposited on the HP-terminated layer increased the *ALPL* level, while it did not increase *RunX2* gene expression in contrast to rhBMP-2 (Figure 7). As mentioned, the most significant effect was obtained as a result of an interplay between HP as a terminal layer, rhBMP-2, and rhBMP-7. There were no significant visible changes in the expression of *OCN* (middle osteogenesis marker).

## 5. Conclusions

The presence of bioactive molecules, i.e., proteins, exposed at the interface between the cell and the material surface influence the cellular response. The proteins, whether deposited on a substrate or present in a solution, regulate cellular functions such as adhesion, migration, and differentiation. In this regard, we have studied the suitability of the two osteogenic growth factors, rhBMP-2 and rhBMP-7, deposited on the (DR/HP)_6_ multilayer system. We proved that these BMPs could be successfully deposited on an HP-terminated layout, under the same conditions, and photochemically bound to the surface. As a result, both proteins separately maintained the osteogenic biological activity, while for the layout composed of the combination of the proteins, the cellular response toward osteoactivity was enhanced. Furthermore, significant differences in cell adhesion and migration were observed: *1)* the HP-terminated layout decreases cell adhesion and migration; *2)* the adsorption of single proteins causes cell elongation and increases cell migration; *3)* combined proteins act additively, eliciting strong cell flattening and increasing the migration rate compared to that on (DR/HP)_6_; however, they show slower cell migration compared to multilayers with single proteins adsorbed. Considering all of these results, our polymeric substrate could be a universal platform for various protein covalent binding. Moreover, the (DR/HP)_6__BMP-2/-7 system may be a promising and versatile coating for bone implants. Strengthening cell adhesion and limiting their mobility after the colonization of the implant site while inducing osteogenesis is a valuable approach to producing new substrates for the needs of bone implantology. The results obtained are in line with the new trend to reduce the doses of these superactive BMPs because of the broad spectrum of drastic adverse effects of current therapies involving supraphysiological amounts of these proteins.

## Figures and Tables

**Table 1:** Results of QCM analyses. The mean mass (calculated from third, fifth, and seventh frequency overtones) of rhBMP-2 and rhBMP-7 deposited on non-photocrosslinked (DR/HP)_6_ layout at different ionic strengths.

Ionic Strength, *I*	Mean Mass (μg/cm^2^)	
	rhBMP-2 (1 μg/mL)	rhBMP-7 (1 μg/mL)
0	2.8	0
1.0 × 10^−3^	3.8	0.3
1.0 × 10^−2^ *	4.4	1.85
0.15	0.8	3.4

* Ionic strength chosen for protein deposition.

## Supplementary Materials

The following supporting information can be downloaded at https://www.mdpi.com/article/10.3390/biom13050842/s1: Figure S1: (A) Heparin structure with marked groups prone to photocrosslinking with DR. Photocrosslinking reaction between diazonium groups of (B) DR and sulfate and (C) carboxyl groups of HP; Figure S2: Release of rhBMP-2 and rhBMP-7 from (DR/HP)_6_ surfaces measured with ELISA assays; Figure S3: Micrographs of hUC-MSC migrating into the scratched cell-free areas taken immediately after “the injury”, after 2, 4, 8, and 12 h of continuous cell migration into the wound. Table S1: Primer list and primer sequences that were used in quantitative real-time PCR analysis of transcripts involved in osteogenic differentiation. F—forward sequence; R—reverse sequence.
