# Supplementary material for: Cellular Response to Bone Morphogenetic Proteins-2 and -7 Covalently Bound to Photocrosslinked Heparin–Diazoresin Multilayer
Source: Biomolecules. 2023 May 15;13(5):842. doi: 10.3390/biom13050842 (PMC10216225; doi:10.3390/biom13050842)
Supplement: Supplementary file 1 [file biomolecules-13-00842-s001.zip › biomolecules-2312805-supplementary.pdf]

Supplementary Materials

Cellular Response to Bone Morphogenetic Proteins-2 and -7 Covalently Bound to Photocrosslinked Heparin–Diazoresin Multilayer

Magdalena Wytrwal, Małgorzata Sekuła-Stryjewska, Agata Pomorska, Ewa Oclon, Ewa Zuba-Surma, Szczepan Zapotoczny and Krzysztof Szczubiałka

1. Covalent bonds formation.

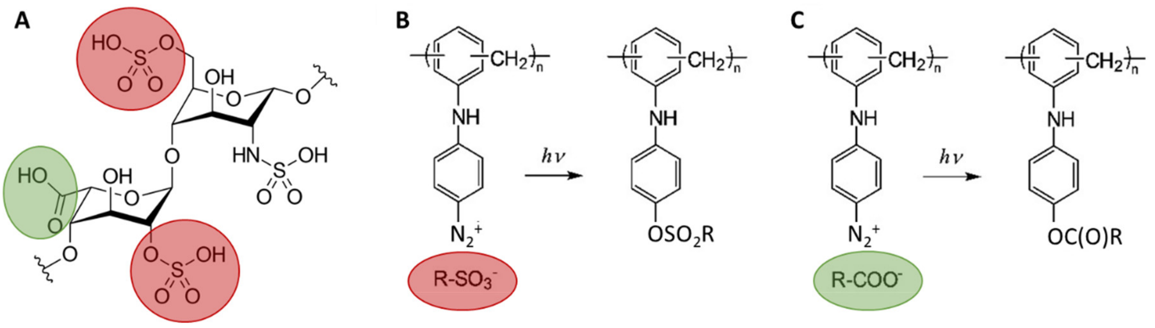

Figure S1. (**A**) Heparin structure with marked groups prone to photocrosslinking with DR. Photocrosslinking reaction between diazonium groups of (**B**) DR and sulfate and (**C**) carboxyl groups of HP.

2. ELISA assay

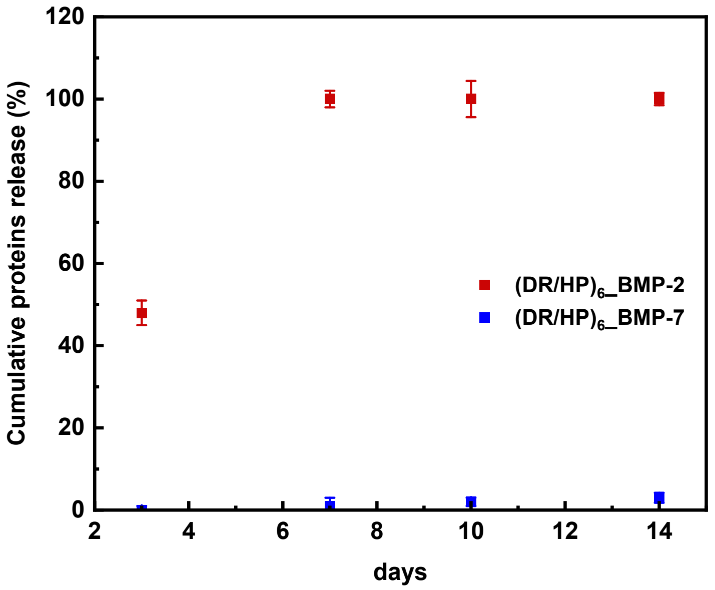

Figure S2. Release of rhBMP-2 and rhBMP-7 from $(DR/HP)_6$ surfaces measured with ELISA assays.

3. Cell migration

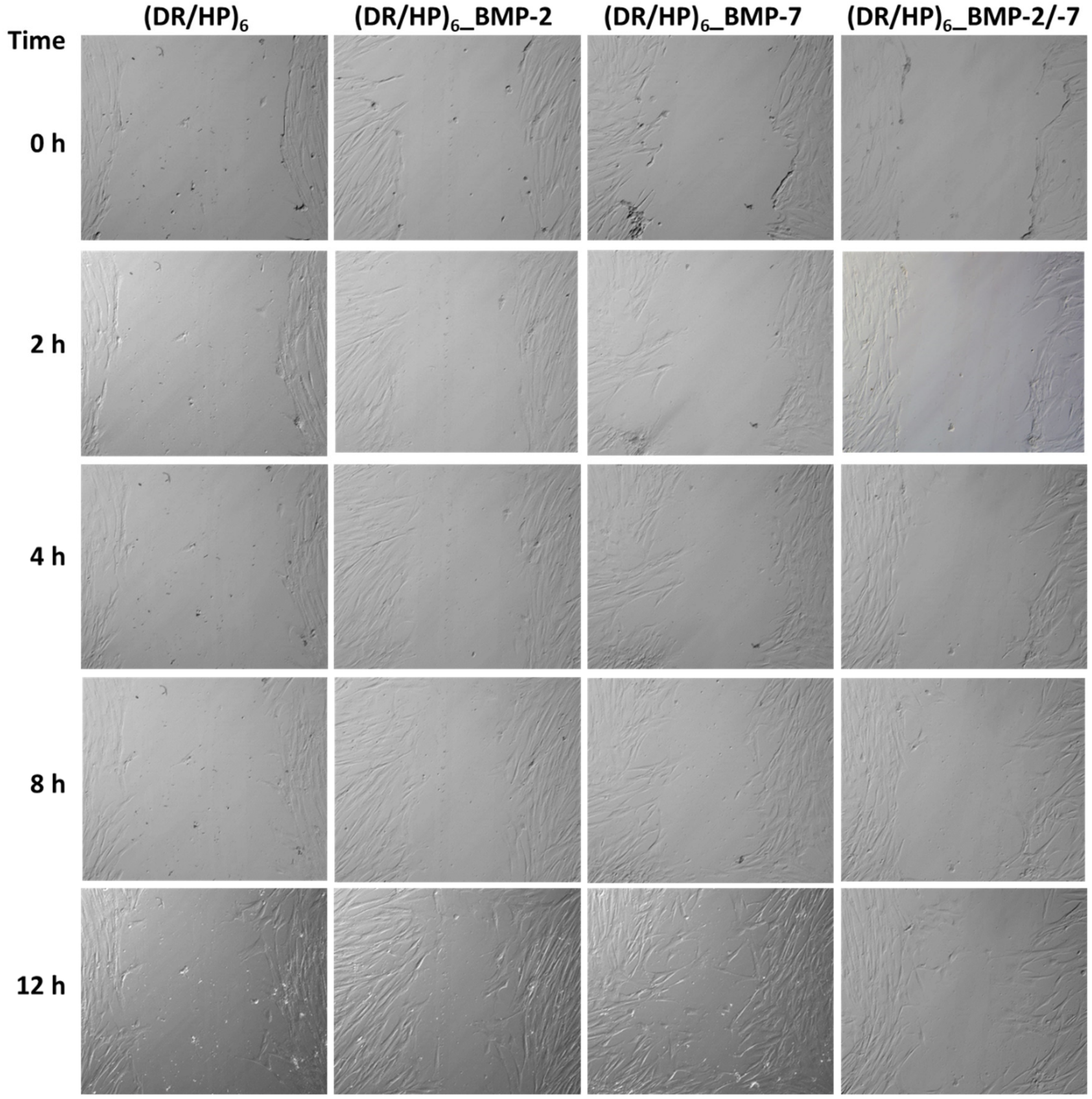

Figure S3. Micrographs of hUC-MSC migrating into the scratched cell-free areas taken immediately after "the injury", after 2, 4, 8 and 12 h of continuous cell migration into the wound.

4. Real-time PCR

Table S1. Primer list and primer sequences that were used in quantitative real-time PCR analysis of transcripts involved in osteogenic differentiation. F - forward sequence; R - reverse sequence.

**Primer Name**	**Sequence**
Human GAPDH -F	CTTTTGCGTCGCCAG
Human GAPDH -R	TTGATGGCAACAATATCCAC
Human ALPL -F	CTATCCTGGCTCCGTGCTCC
Human ALPL -R	TTAACTGATGTTCCAATCCTGCG
Human OCN -F	GACTGTGACGAGTTGGCTGA
Human OCN -R	CTGGAGAGGAGCAGAACTGG
Human RunX2 -F	GGAGTGGACGAGGCAAGAGTTT
Human RunX2 -R	AGCTTCTGTCTGTGCCTTCTGG
